# Quantum phases for moving charges and dipoles in an electromagnetic field and fundamental equations of quantum mechanics

**DOI:** 10.1038/s41598-018-30423-8

**Published:** 2018-08-09

**Authors:** A. L. Kholmetskii, T. Yarman, O. V. Missevitch, M. Arik

**Affiliations:** 1 Department of Physics, Belarus State University, 4 Nezavisimosti Avenue, 220030 Minsk, Belarus; 2 Department of Engineering, Okan University, Akfirat, Istanbul, Turkey & Savronik, Eskisehir, Turkey; 3 Institute for Nuclear Problems, Belarus State University, 11 Bobruiskaya Str., 220030 Minsk, Belarus; 4 Bogazici University, Istanbul, Turkey

## Abstract

We analyze the quantum phase effects for point-like charges and electric (magnetic) dipoles under a natural assumption that the observed phase for a dipole represents the sum of corresponding phases for charges composing this dipole. This way we disclose two novel quantum phases for charged particles, which we named as complementary electric Aharonov-Bohm (A-B) phase and complementary magnetic A-B phase, respectively. We reveal that these phases are derived from the Schrödinger equation only in the case, where the operator of momentum is re-defined via the replacement of the canonical momentum of particle by the sum of its mechanical momentum and interactional field momentum for a system of charged particles. The related alteration should be introduced to Klein-Gordon and Dirac equations, too, and implications of this modification are discussed.

## Introduction

It is known that the Schrödinger equation with the standard Hamiltonian for a charged particle in an electromagnetic (EM) field

1 $$\hat{H}=\frac{{(-i\hslash \nabla -e{\boldsymbol{A}}/c)}^{2}}{2m}+e\phi $$

yield two quantum phase effects with the electric Aharonov-Bohm (A-B) phase^1^

2a $${\delta }_{\phi }=\frac{e}{\hslash }\int \phi dt,$$

and the magnetic A-B phase^1^

2b $${\delta }_{A}=\frac{e}{\hslash c}\int {\boldsymbol{A}}\cdot d{\boldsymbol{s}},$$

respectively, where common notations are used. In particular, *φ* is the scalar potential, ***A*** is the vector potential, *d****s*** = ***v****dt* is the path element of a charged particle *e*, and ***v*** is its velocity. We emphasize that no other quantum phase effects for point-like charges could be derived with the Hamiltonian (1).

After the publications^1,2^ it was realized that quantum phase effects exist for moving dipoles, too, and the first such effect had been predicted by Aharonov and Casher for a magnetic dipole moving in an electric field ***E***^3^, with the A-C phase

3 $${\delta }_{mE}=\frac{1}{\hslash c}\int ({{\boldsymbol{m}}}_{0}\times {\boldsymbol{E}})\cdot d{\boldsymbol{s}},$$

***m***_0_ being the proper magnetic dipole moment.

Later, a similar quantum phase effect had been predicted for the electric dipole ***p***_0_, moving in the magnetic field ***B***, characterized by the He-McKellar-Wilkens (HMW) phase^4,5^

4 $${\delta }_{pB}=-\,\frac{1}{\hslash c}\int ({{\boldsymbol{p}}}_{0}\times {\boldsymbol{B}})\cdot d{\boldsymbol{s}}.$$

We emphasize that both phases (3) and (4) have been discovered experimentally (e.g.^6,7^). However, at that time it was unclear, whether eqs (3), (4) give all possible phases for moving dipoles, because a commonly recognized expression for the Hamiltonian of electric/magnetic dipole in an EM field did not exist; moreover, even in the classical limit, a consensus with respect to the force on a dipole was not achieved up to the modern time (see, e.g.^8–12^).

Recently, using the known expression for the Lagrangian density $L={M}^{\alpha \beta }{F}_{\alpha \beta }/2$ in material media (where *M*^*αβ*^ is the magnetization-polarization tensor, and *F*^*αβ*^ is the tensor of EM field) and integrating it to a compact dipole, we explicitly determined the relativistic motional equation for a dipole and its Hamiltonian^13^. The corresponding expression for the quantum phase reads

5 $$\begin{array}{rcl}\delta  & = & \frac{1}{\hslash c}\int ({{\boldsymbol{m}}}_{0}\times {\boldsymbol{E}})\cdot d{\boldsymbol{s}}-\frac{1}{\hslash c}\int ({{\boldsymbol{p}}}_{0}\times {\boldsymbol{B}})\cdot d{\boldsymbol{s}}\\  &  & -\frac{1}{\hslash {c}^{2}}\int \gamma ({{\boldsymbol{p}}}_{0//}\cdot {\boldsymbol{E}}){\boldsymbol{v}}\cdot d{\boldsymbol{s}}-\frac{1}{\hslash {c}^{2}}\int \gamma ({{\boldsymbol{m}}}_{0//}\cdot {\boldsymbol{B}}){\boldsymbol{v}}\cdot d{\boldsymbol{s}}\\  &  & -\frac{1}{\hslash }\int ({\boldsymbol{p}}\cdot {\boldsymbol{E}})dt-\frac{1}{\hslash }\int ({\boldsymbol{m}}\cdot {\boldsymbol{B}})dt,\end{array}$$

where two novel phase effects emerge,

6 $${\delta }_{pE}=-\,\frac{1}{\hslash {c}^{2}}\int \gamma ({{\boldsymbol{p}}}_{0//}\cdot {\boldsymbol{E}}){\boldsymbol{v}}\cdot d{\boldsymbol{s}},$$

7 $${\delta }_{mB}=-\,\frac{1}{\hslash {c}^{2}}\int \gamma ({{\boldsymbol{m}}}_{0//}\cdot {\boldsymbol{B}}){\boldsymbol{v}}\cdot d{\boldsymbol{s}},$$

next to the phases (3), (4), being described by the first and second terms of eq. (5). Here $\gamma ={(1-{v}^{2}/{c}^{2})}^{-1/2}$, and the subscript “//” denotes the vector component collinear with ***v***. The emergence of factor *γ* in eqs (5–7) is explained by the specific dependence of the electric (magnetic) dipole moment on ***v***. Indeed, for a pure electric dipole with a vanishing proper magnetic dipole moment, we can write ${\boldsymbol{p}}={\gamma }^{-1}{{\boldsymbol{p}}}_{0//}+{{\boldsymbol{p}}}_{0\perp }$. This equation indicates that the component of electric dipole moment ${{\boldsymbol{p}}}_{0\perp }$, which is orthogonal to vector ***v***, does not depend on ***v***. Hence, the canonical momentum of the electric dipole $\partial L/\partial {\boldsymbol{v}}$ does not depend on ${{\boldsymbol{p}}}_{0\perp }$, and contains the term proportional to $\frac{\partial }{\partial {\boldsymbol{v}}}({\gamma }^{-1}{{\boldsymbol{p}}}_{0//}\cdot {\boldsymbol{E}})=-\gamma ({{\boldsymbol{p}}}_{0//}\cdot {\boldsymbol{E}}){\boldsymbol{v}}/{c}^{2}$ (see eq. (6)). Similar reasoning is applicable to the magnetic dipole, see eq. (7).

We add that the last two integrals in eq. (5) determine respectively the Stark phase^14^ and Zeeman phase^15^, which do not explicitly depend on the velocity of dipole, and are excluded from further analysis.

We point out that the phases (3), (4) and (6), (7) correspond to all possible combinations of the pair ***p***, ***m*** with the pair ***E***, ***B***, and are related via electric-magnetic duality transformations ***E*** → ***B***, ***p*** → ***m***^16^.

In our analysis of section 2, aimed to determine the physical meaning of quantum phases (3), (4), (6), (7), we consider a dipole as a compact electrically neutral bunch of point-like charges. We further adopt that these phases represent a superposition of the appropriate phase effects for each charge of the bunch. In other words, we introduce quantum phase superposition principle based on superposition principle for EM field. Using this idea, we find two novel phase effects for point-like charges,

8 $${\delta }_{c\phi }=-\,\frac{1}{\hslash {c}^{2}}\int e\phi {\boldsymbol{v}}\cdot d{\boldsymbol{s}},$$

9 $${\delta }_{cA}=-\,\frac{e}{\hslash {c}^{3}}\int ({\boldsymbol{v}}\cdot {\boldsymbol{A}}){\boldsymbol{v}}\cdot d{\boldsymbol{s}},$$

named as the complementary electric (*δ*_*cφ*_) and the complementary magnetic (*δ*_*cA*_) A-B phase, correspondingly, which explain the origin of *δ*_*mE*_, *δ*_*pE*_, *δ*_*mB*_ phases at the fundamental level.

In section 3 we emphasize that the phases (8) and (9) do not result from the Schrödinger equation with the standard Hamiltonian (1). We further reveal that the phases (8), (9) emerge in the case, where the operator of momentum for charged particle in an EM field is re-defined as the sum of its mechanical momentum and interactional field momentum, instead of the standard definition of this operator via the canonical momentum of charged particle.

This result implies that the same re-definition of the operator of momentum should be made in Klein-Gordon and Dirac equations as well, and in section 4 we discuss possible implications of this step. Finally, we conclude in section 5.

## Origin of Quantum Phase Effects for Electric/magnetic Dipoles

In this section we adopt for simplicity the weak relativistic limit, corresponding to the accuracy of calculations *c*^−2^ for electric effects and *c*^−3^ for magnetic effects. As is shown in refs^17,18^, in this limit the first four terms of eq. (5) can be presented in the convenient form

10 $$\delta \approx \frac{1}{\hslash c}\int ({\boldsymbol{m}}\times {\boldsymbol{E}})\cdot d{\boldsymbol{s}}-\frac{1}{\hslash c}\int ({\boldsymbol{p}}\times {\boldsymbol{B}})\cdot d{\boldsymbol{s}}-\frac{1}{\hslash {c}^{2}}\int ({\boldsymbol{p}}\cdot {\boldsymbol{E}}){\boldsymbol{v}}\cdot d{\boldsymbol{s}}-\frac{1}{\hslash {c}^{2}}\int ({\boldsymbol{m}}\cdot {\boldsymbol{B}}){\boldsymbol{v}}\cdot d{\boldsymbol{s}},$$

where all quantities are defined in a labframe.

We want to understand, how eq. (10) is related to the phase effects for point-like charges. Exploring this problem, we emphasize that the electric A-B phase (2a), being not explicitly dependent on ***v***, can explain none of the phases in eq. (10). As is known, the magnetic A-B phase (2b) explains the HMW phase only (the second term of eq. (10))^17,19^. Therefore, one can conjecture the existence of more quantum phase effects for point-like charges (next to the A-B phases (2a-b)), required to explain all terms of eq. (10).

Determining corresponding phases, we use the appropriate models of electric and magnetic dipoles. In particular, adopting the simplest model of electric dipole (two point-like charges −*e* and +*e* separated by a small distance ***d***), we find^17,18^ that the third term of eq. (10) can be presented in the form

11 $${\delta }_{pE}=-\,\frac{1}{\hslash {c}^{2}}\int ({\boldsymbol{p}}\cdot {\boldsymbol{E}}){\boldsymbol{v}}\cdot d{\boldsymbol{s}}=-\,\frac{1}{\hslash {c}^{2}}{\int }_{V}\oint \rho \phi {\boldsymbol{v}}\cdot d{\boldsymbol{s}}dV=-\,\frac{1}{\hslash {c}^{2}}\oint (e\phi ({\boldsymbol{r}}+{\boldsymbol{d}})-e\phi ({\boldsymbol{r}})){\boldsymbol{v}}\cdot d{\boldsymbol{s}}.$$

This equation shows that the phase *δ*_*pE*_ for a moving electric dipole in the presence of electric field represents the superposition of fundamental quantum phases for each charge of the dipole, defined by eq. (8). The phase (8) depends on the scalar potential *φ*, and in the weak relativistic limit, it is (*v*/*c*)^2^ smaller than the electric A-B phase (2a). We named the phase (8) as the complementary electric A-B phase and found^17^ that it is also responsible for the A-C phase (the first term of eq. (10)) due to the equality

12 $${\delta }_{mE}=\frac{1}{\hslash c}\int ({\boldsymbol{m}}\times {\boldsymbol{E}})\cdot d{\boldsymbol{s}}=-\,\frac{1}{\hslash {c}^{2}}{\int }_{V}\oint \phi \rho {\boldsymbol{u}}\cdot d{\boldsymbol{s}}dV,$$

derived in^17^ for a magnetic dipole, considered as a small conducting loop with the charge density of carriers of current *ρ* and their flow velocity ***u***. Then one can see that the phase (12) represents the algebraic sum of fundamental phases (8) for each carries of current in the dipole.

Finally, for the last term on rhs of eq. (10), we derive^17,18^

13 $${\delta }_{mB}=-\,\frac{1}{\hslash {c}^{2}}\int ({\boldsymbol{m}}\cdot {\boldsymbol{B}}){\boldsymbol{v}}\cdot d{\boldsymbol{s}}=-\,\frac{1}{\hslash {c}^{3}}{\int }_{V}\int ({\boldsymbol{j}}\cdot {\boldsymbol{A}}){\boldsymbol{v}}\cdot d{\boldsymbol{s}}dV,$$

where ***j*** is the current density of carriers of current in the magnetic dipole ***m***. We clarify the origin of the phase effect (13) for the charge *e*, moving along a circular orbit at some velocity ***v*** with the current density ${\boldsymbol{j}}=\delta ({\boldsymbol{r}}-{{\boldsymbol{r}}}_{0})e{\boldsymbol{v}}$. Substituting the latter equation into eq. (13), we arrive at eq. (9), which thus defines one more fundamental quantum phase for a point-like charge moving in the field ***A***, named in ref.^17^ as the complementary magnetic A-B phase.

**Figure 1:**
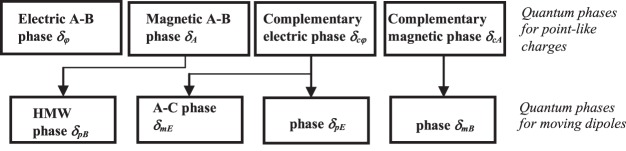
Relationship between quantum phases for charged particles and for moving dipoles.

Thus, we demonstrated that each term of eq. (10) derived for a moving dipole finds its physical interpretation via the corresponding fundamental phase for point-like charge, as is shown in Fig. 1 adapted from refs^17,18^.

We see that the electric A-B phase (2a) does not contribute to quantum phases for moving dipoles. This is explained by the fact that the phases for dipoles *δ*_*pB*_, *δ*_*mE*_, *δ*_*pE*_, *δ*_*mB*_, are path-dependent, whereas the electric A-B phase (2a) does not explicitly depend on ***v***.

## Quantum Phases and Hamiltonian of Charged Particle in an Electromagnetic Field

As we emphasized above, the standard Hamiltonian (1) yields the magnetic (2a) and electric (2b) A-B phases only, and the present disclosure of the complementary electric (8) and magnetic (9) A-B phases for point-like charges indicates the presence of inconsistencies in quantum description of charged particles in an EM field. In our opinion, an inconsistency is related to the physical interpretation of the operator of momentum of charged particle $\hat{{\boldsymbol{p}}}=-\,i\hslash \nabla$. It is known that in the presence of EM field, this operator is associated with the canonical momentum ***P***_*c*_ of particle

14 $${{\boldsymbol{P}}}_{c}={\boldsymbol{p}}+\frac{e{\boldsymbol{A}}}{c}\to {\hat{{\boldsymbol{P}}}}_{c}=-\,i\hslash \nabla ,$$

which straightforwardly leads to eq. (1).

In fact, equation (14) prescribes the fundamental role to the canonical momentum of particle; therefore, it is important to clarify its physical meaning, in particular, with respect to the term *e****A***/*c*.

In ref.^18^ we demonstrate that the term *e****A***/*c* describes the momentum of interactional EM field ***P***_*EM*_ for a system “charged particle and a source of external EM field” in the particular case, where the particle *rests* in the laboratory frame, i.e.

15 $${{\boldsymbol{P}}}_{EM}(v=0)=e{\boldsymbol{A}}/c.$$

This means that the canonical momentum ***P***_*c*_ (14) is composed as the sum of mechanical momentum of *moving* particle ***p*** and interactional field momentum (15), when the particle *is at rest* in a laboratory frame. This finding clearly indicates that ***P***_*c*_ does not have a real physical meaning, and it emerges only as a formal variable in the classical Lagrangian formalism.

Therefore, in the quantum domain it looks natural to define the operator of momentum via the sum of mechanical momentum of particle ***p*** and the interactional field momentum ***P***_*EM*_ for a *moving* charge. This suggests re-postulating the operator of momentum as

16 $${\boldsymbol{p}}+{{\boldsymbol{P}}}_{EM}\to \hat{{\boldsymbol{P}}}=-\,i\hslash \nabla .$$

In this case, the Hamiltonian (1) is replaced by

17 $$\hat{H}=\frac{{(-i\hslash \nabla -{{\boldsymbol{P}}}_{EM})}^{2}}{2m}+e\phi .$$

For a free spinless charged particle in the external ***E***, ***B*** fields, the explicit expression for ***P***_*EM*_ via the field potentials reads as^18^

18 $${{\boldsymbol{P}}}_{EM}=\frac{e{\boldsymbol{A}}}{c}+\frac{{\boldsymbol{v}}e\phi }{{c}^{2}}+\frac{e{\boldsymbol{v}}({\boldsymbol{A}}\cdot {\boldsymbol{v}})}{{c}^{3}},$$

Substituting eq. (18) into eq. (17), we obtain

19 $$H=\frac{1}{2m}{(-i\hslash \nabla -\frac{e{\boldsymbol{A}}}{c}-\frac{{\bf{v}}e\phi }{{c}^{2}}-\frac{e{\boldsymbol{v}}({\boldsymbol{A}}\cdot {\boldsymbol{v}})}{{c}^{3}})}^{2}+e\phi ,$$

where all variables are understood as operators.

The quantum phase for a point-like charge in an EM field is defined by the equation^2^

20 $$\delta =\frac{1}{\hslash }\int (H-{H}_{0})dt,,$$

where *H*_0_ stands for the Hamiltonian of particle for a vanishing EM field. Combining (19), (20), and using the Coulomb gauge, where the operators ***v*** and ***A*** commutate with each other, we arrive at the following expression for the total quantum phase:

21 $$\delta =\frac{1}{\hslash }\int e\phi dt-\frac{1}{\hslash c}\int e{\boldsymbol{A}}\cdot d{\boldsymbol{s}}-\frac{1}{\hslash {c}^{2}}\int e\phi {\boldsymbol{v}}\cdot d{\boldsymbol{s}}-\frac{1}{\hslash {c}^{3}}\int e({\boldsymbol{A}}\cdot {\boldsymbol{v}}){\boldsymbol{v}}\cdot d{\boldsymbol{s}},$$

Here we neglected the terms, containing the mass *m* in their denominators, which is warranted in any practical situation.

One can see that the first and the second terms of eq. (21) determine the electric (2a) and magnetic (2b) A-B phases, while the third and fourth terms define the complementary electric (8) and complementary magnetic (9) A-B phases, correspondingly.

Thus, having defined the operator of momentum according to eq. (16), we achieved a full harmony between eqs (21) and (10), describing quantum phase effects for charges and dipoles.

## Re-defined Operator of Momentum in the Dirac Equations

The disclosure of complementary electric (8) and magnetic (9) A-B phases, which are proportional to *c*^−2^ and *c*^−3^, correspondingly, suggests that their consistent description should be done via the Klein-Gordon equation (for spinless particles), or via the Dirac equation (for electrons), with the operator of momentum defined by eq. (16). More specifically, in the presence of EM field, the operator ${\partial }^{\mu }$ in the Klein-Gordon equation should be replaced by ${\partial }^{\mu }-{{P}_{EM}}^{\mu }$ (*μ* = 0…3), ${{P}_{EM}}^{\mu }$ being the four-momentum for interactional EM field. The implications of this modification of the Klein-Gordon equation will be considered elsewhere; here we focus our attention to the Dirac equation, where the re-definition (16) of the momentum operator leads to the Hamiltonian

22 $$H={\boldsymbol{\alpha }}(-i\hslash \nabla -{{\boldsymbol{P}}}_{EM})+\beta m{c}^{2}+{U}_{el}.$$

Here $$\alpha =(\begin{array}{cc}0 & {\boldsymbol{\sigma }}\\ {\boldsymbol{\sigma }} & 0\end{array})$$ (***σ*** being the Pauli matrix), $$\beta =(\begin{array}{cc}{\bf{1}} & 0\\ 0 & -{\bf{1}}\end{array})$$, and we designated the electric interaction energy ${U}_{el}=e\phi$, which is convenient in further analysis.

It is known that the Dirac equation with the Hamiltonian (22) leads to the Pauli equation in the weak relativistic limit. Thus, for a *free* electron moving in an EM field with the positive total energy, due to equation (18), we again derive complementary electric (8) and complementary magnetic (9) phases, next to the A-B phases (2a-b). Taking into account that the electron possesses the magnetic dipole moment, we also get the phases (3), (7) for magnetic dipole.

When the total energy of electron in an EM field is negative (the *bound* electron), eq. (18) becomes inapplicable for two reasons. First, for bound charges, the approximation of constancy of ***E***, ***B*** in a vicinity of each charge (used in the derivation of eq. (18)) is no longer fulfilled. Next reason, which makes eq. (18) incorrect for bound electron, has the fundamental origin and is related to the known fact that an electrically bound system in the stationary energy state does not radiate. Therefore, the standard Maxwell equations used in the derivation of eq. (18) become inapplicable, insofar as their solution includes both the non-radiating (bound) and radiating field components.

In ref.^20^, we already proposed a modification of Maxwell equations with the elimination of radiative field component, which keeps their Lorentz-invariance:

23a-d $$\nabla \cdot {\boldsymbol{E}}=4\pi \rho ,\nabla \cdot {\boldsymbol{B}}=0,\nabla \times {\boldsymbol{E}}=-\,\frac{1}{c}({\boldsymbol{v}}\cdot \nabla ){\boldsymbol{B}},\,\nabla \times {\boldsymbol{B}}=\frac{1}{c}({\boldsymbol{v}}\cdot \nabla ){\boldsymbol{E}}+\frac{4\pi {\boldsymbol{j}}}{c}.$$

Here ***v*** is some effective velocity parameter, which for the electron in the *s*-state has equal spatial components, and its modulus coincides with the modulus of averaged velocity of the electron. In fact, the structure of eqs (23a–d) corresponds to the Maxwell equations for a charged particle, moving with a constant velocity. It is known that the EM field generated by such particle is described by the Heaviside solution^16^, which does not contain radiative component.

Next, using eq. (23), we determine the momentum of interactional EM field ***P***_*EM*_ for the simplest one-body problem, where the electron is bound to a heavy immovable nucleus with the positive charge *Ze*. Then, in the semi-classical limit we obtain

24 $${{\boldsymbol{P}}}_{EM}=\frac{1}{4\pi c}{\int }_{V}({\boldsymbol{E}}\times {{\boldsymbol{B}}}_{e})dV=\frac{1}{4\pi c}{\int }_{V}({\boldsymbol{E}}\times (\nabla \times {{\boldsymbol{A}}}_{e}))dV.$$

Using the identity^21^

$${\int }_{V}({\boldsymbol{E}}\times (\nabla \times {{\boldsymbol{A}}}_{e}))dV+{\int }_{V}({{\boldsymbol{A}}}_{e}\times (\nabla \times {\boldsymbol{E}}))dV-{\int }_{V}({\boldsymbol{E}}(\nabla \cdot {{\boldsymbol{A}}}_{e}))dV-{\int }_{V}({{\boldsymbol{A}}}_{e}(\nabla \cdot {\boldsymbol{E}}))dV=0,$$

and involving the equalities $\nabla \times {\boldsymbol{E}}=0$, $\nabla \cdot {\boldsymbol{E}}=4\pi \rho$ (for immovable host charge) along with the Coulomb gauge $\nabla \cdot {{\boldsymbol{A}}}_{e}=0$, we derive

25 $${\int }_{V}({\boldsymbol{E}}\times (\nabla \times {{\boldsymbol{A}}}_{e}))dV=4\pi {\int }_{V}{{\boldsymbol{A}}}_{e}\rho dV=\frac{{{\boldsymbol{A}}}_{e}Ze}{c}.$$

According to the Heaviside solution, the vector potential of the electron at the location of host charge is equal to ${{\boldsymbol{A}}}_{e}=\gamma e{\boldsymbol{v}}/rc$, where *r* is the classical radius of electron’s orbit, and *γ* is its Lorentz factor. Hence, combining eqs (24), (25), we obtain

26 $${{\boldsymbol{P}}}_{EM}=\frac{1}{4\pi c}{\int }_{V}({\boldsymbol{E}}\times {{\boldsymbol{B}}}_{e})dV=\gamma \frac{e\phi {\boldsymbol{v}}}{{c}^{2}},$$

where $\phi =Ze/r$ is the scalar potential of host charge at the location of the electron.

Substituting (26) into (16), and taking into account the equality ${\boldsymbol{p}}=\gamma m{\boldsymbol{v}}$, we reveal that for the one-body problem, the momentum operator is defined by the relationship

27 $$\gamma m{\boldsymbol{v}}+\gamma \frac{e\phi {\boldsymbol{v}}}{{c}^{2}}=\gamma m(1+\frac{e\phi }{m{c}^{2}}){\boldsymbol{v}}=\gamma mb\to \hat{{\boldsymbol{P}}}=-\,i\hslash \nabla ,$$

where we introduced the “binding” factor

28 $$b=(1+e\phi /m{c}^{2}).$$

Thus, we recover the standard definition of the operator of momentum via the replacement of the rest mass *m* by *bm*, i.e.

29 $$m\to bm.$$

Next, we determine the electric interactional energy *U*_*el*_ with the field equation (23). Omitting straightforward calculations, which are based on the Heaviside solution for the fields/potentials, we present the final result ${U}_{el}=\gamma e\phi$. This means that in comparison with the free electron, we get the replacement

30 $$e\phi \to \gamma e\phi .$$

Correspondingly, the Hamiltonian acquires the form

31 $$H=-\,{\boldsymbol{\alpha }}i\hslash \nabla +\beta bm{c}^{2}+\gamma {U}_{el}.$$

The replacements (29), (30) for the one-body problem had been proposed for the first time by Yarman^22^, and later derived in our paper^20^ within the purely bound field constraint. In particular, we have shown in^20^ that these replacements do not change the solution of the Dirac-Coulomb equation and thus do not affect the fine structure of the atomic energy levels. However, important corrections do emerge at hyperfine level.

The analysis of hyperfine contributions to the energy levels of hydrogenlike atoms requires considering the two-body problem for bound particles with the rest masses *m* and *M*, correspondingly. Re-definition of the momentum operator according to eq. (16) for two-body problem leads to replacements of eqs (29), (30) by

32a-c $$m\to m{b}_{m},M\to M{b}_{M},U\to {\gamma }_{mn}{\gamma }_{Mn}U,$$

with

33a-d $${b}_{m}=(1+\frac{{\gamma }_{M}U}{m{c}^{2}}),{b}_{M}=(1+\frac{{\gamma }_{m}U}{M{c}^{2}}),\,{\gamma }_{m}={(1-{{v}_{m}}^{2}/{c}^{2})}^{-1/2},\,{\gamma }_{M}={(1-{{v}_{M}}^{2}/{c}^{2})}^{-1/2},$$

in the same way as in^20^.

The theory, where the substitutions (32a–c) are applied to the precise physics of light hydrogenlike atoms, had been named in^20^ as the Purely Bound Field Theory (PBFT). It has been shown in the subsequent publication^23^ that the application of PBFT to this area of physics allows eliminating all available deviations between theory and experiment, and the most principal achievements of PBFT are:

- The correction of QED result with respect to the 1*S*-2*S* interval in positronium from $${E}_{1S-2S}^{Ps}({\rm{QED}})=$$$$1\,233\,607\,222.2(6)\,{\rm{MHz}}$$^24^ to $${E}_{1S-2S}^{Ps}({\rm{PBFT}})=1\,233\,607\,214.7(6)\,{\rm{MHz}}$$^23^ (the measurement result is $${E}_{1S-2S}^{Ps}=1\,233\,607\,216(3)\,{\rm{MHz}}$$^25^);
- The correction of QED result with respect to the 1 S spin-spin interval in positronium from $$W{({\rm{QED}})}_{s-s}^{Ps}=$$$$203\,391.7(6)\,{\rm{MHz}}$$^24^ to $$W{({\rm{PBFT}})}_{s-s}^{Ps}=203\,386(1)\,{\rm{MHz}}$$^23^ (the measurement result is $${W}_{s-s}^{Ps}=203\,387(2)$$ MHz)^26^);
- The derivation of the proton charge radius via the 2*S*-2*P* Lamb shift in hydrogen (0.841(6) fm^27^) and via the 1S Lamb shift in the hydrogen (0.846(22) fm^27^) in a full agreement with the recent result derived for muonic hydrogen (0.84087(39) fm^28^).

We add that for any other problem of precise physics of hydrogen-like atoms, where the agreement between experimental data and QED calculations has been achieved before creation of PBFT, the deviation between the results of QED and PBFT is less than the measurement precision^23^. Nevertheless, up to date re-scaling of rest masses according to eq. (32a-b) looked somewhat artificial. Now we see that this modification of rest masses is directly related to the proposed re-definition of the momentum operator (16), and acquires a deep physical meaning. This circumstance substantially enriches the physical content of PBFT.

## Conclusion

The disclosure of the novel quantum phase effects for point-like charges, named as complementary electric A-B phase (8) and complementary magnetic A-B phase (9), requires to re-define the operator of momentum for charged particle in an EM field according to eq. (16), in order to include these phases into the solution of the Schrödinger equation.

It is important to stress that the complementary electric phase (8) is directly responsible for the A–C effect due to eq. (12) and thus, the experimental confirmation of the A–C effect (see, e.g.,^6^) serves, in fact, as the proof of validity of re-definition (16).

The suggested re-definition of the momentum operator (16) acquires the fundamental significance for electrically bound charges, where we additionally have to take into account the non-radiative origin of EM fields generated by such charges. In this way we fully eliminate the available subtle deviations between the results of QED calculations and experimental data in precise physics of simple atoms.

### Data availability statement

No datasets were generated or analyzed during the current study.
